# Direct Antibiotic Activity of Bacillibactin Broadens the Biocontrol Range of Bacillus amyloliquefaciens MBI600

**DOI:** 10.1128/mSphere.00376-21

**Published:** 2021-08-11

**Authors:** Anastasia Dimopoulou, Ioannis Theologidis, Dimitra Benaki, Marilena Koukounia, Amalia Zervakou, Aliki Tzima, George Diallinas, Dimitris G. Hatzinikolaou, Nicholas Skandalis

**Affiliations:** a Institute of Molecular Biology and Biotechnology, FORTH, Heraklion, Greece; b Laboratory of Pesticides’ Toxicology, Benaki Phytopathological Institute, Athens, Greece; c Department of Pharmacy, National and Kapodistrian University of Athens, Athens, Greece; d Department of Biology, Sector of Botany, National and Kapodistrian University of Athens, Athens, Greece; e Laboratory of Plant Pathology, Agricultural University of Athens, Athens, Greece; f Department of Medicine, Keck School of Medicine, University of Southern California, Los Angeles, California, USA; University of California, Davis

**Keywords:** *Bacillus amyloliquefaciens*, siderophores, antibiotics, iron starvation, plant pathogens, bactericides, plant-pathogenic bacteria

## Abstract

Bacillus amyloliquefaciens is considered the most successful biological control agent due to its ability to colonize the plant rhizosphere and phyllosphere where it outgrows plant pathogens by competition, antibiosis, and inducing plant defense. Its antimicrobial function is thought to depend on a diverse spectrum of secondary metabolites, including peptides, cyclic lipopeptides, and polyketides, which have been shown to target mostly fungal pathogens. In this study, we isolated and characterized the catecholate siderophore bacillibactin by *B. amyloliquefaciens* MBI600 under iron-limiting conditions and we further identified its potential antibiotic activity against plant pathogens. Our data show that bacillibactin production restrained *in vitro* and *in planta* growth of the nonsusceptible (to MBI600) pathogen Pseudomonas syringae pv. *tomato*. Notably, it was also related to increased antifungal activity of MBI600. In addition to bacillibactin biosynthesis, iron starvation led to upregulation of specific genes involved in microbial fitness and competition.

**IMPORTANCE** Siderophores have mostly been studied concerning their contribution to the fitness and virulence of bacterial pathogens. In the present work, we isolated and characterized for the first time the siderophore bacillibactin from a commercial bacterial biocontrol agent. We proved that its presence in the culture broth has significant biocontrol activity against nonsusceptible bacterial and fungal phytopathogens. In addition, we suggest that its activity is due to a new mechanism of action, that of direct antibiosis, rather than by competition through iron scavenging. Furthermore, we showed that bacillibactin biosynthesis is coregulated with the transcription of antimicrobial metabolite synthases and fitness regulatory genes that maximize competition capability. Finally, this work highlights that the efficiency and range of existing bacterial biocontrol agents can be improved and broadened via the rational modification of the growth conditions of biocontrol organisms.

## INTRODUCTION

Iron is a vital element required by every living organism that functions as a cofactor in numerous cellular processes, such as the tricarboxylic acid cycle, electron transport chain, and oxidative phosphorylation, or the biosynthesis of vitamins, antibiotics, toxins, and other Fe-containing aromatic compounds (1). It also plays an important role in microbial biofilm formation by regulating the surface motility of microorganisms (2, 3). Under certain environmental conditions, the level of physiologically available concentrations of iron can drop far below 1 mM (4). As a survival strategy, many bacteria, fungi, and monocotyledonous plants synthesize and secrete high-affinity extracellular siderophores to acquire iron (5, 6). Additional functions of bacterial siderophores include the alteration of the soil microbial community (7), the promotion of plant growth (8), the potential use as biocontrol agents (9, 10), and the enhancement of bioremediation of heavy metals (11, 12). Siderophores have also been exploited as biosensors (13, 14) and as selective mediators of antibiotic clinical bacteria (Trojan horse strategy) (15).

Siderophores are low-molecular-weight molecules in the range of approximately 400 to 1,500 Da. They usually form hexadentate or octahedral complexes with ferric iron and typically employ hydroxamates, α-hydroxycarboxylates and catechols as extremely effective Fe^3+^ ligands; thus, they can be classified as hydroxamate, hydroxycarboxylate, or catecholate type siderophores, respectively (16). The hydroxamate-type siderophores are produced by Pseudomonas spp. (17), while the catecholate types are produced by Gram-negative Escherichia coli, Salmonella enterica serovar Typhimurium, and Klebsiella pneumonia, which produce enterochelin (18), and Gram-positive *Bacillus* spp. that produce bacillibactin (19).

*Bacillus* species dominate the biopesticide market (20). Among them, *B. amyloliquefaciens* is an exceptionally pervasive species due to its ability to colonize different ecological niches such as soil, water, a variety of surfaces, and the rhizospheres of many plants (21). This adaptation in a wide range of environments derives from its impressive ability to produce a diverse spectrum of antagonistic secondary metabolites, including peptides, cyclic lipopeptides, and polyketides. Such peptides can facilitate root colonization and interaction with the host plant and prime plant defense responses (22, 23).

*B. amyloliquefaciens* has been considered the most competitive strain among biological control agents (BCAs) based on its antifungal activity against agronomically important fungal phytopathogens (24–26). However, little is known about its bactericidal activity and mode of action. *B. amyloliquefaciens* has been reported to control a few economically important bacterial diseases, such as the bacterial blight and bacterial leaf streak of rice caused by Xanthomonas oryzae (27), fire blight disease (Erwinia amylovora) (28), and tomato wilting (Ralstonia solanacearum) (29). More recent studies have shown biocontrol activity against some Pseudomonas syringae pathovars on sugar beet (30) and tomato plants (31, 32). Serifel is a commercial formulation of Bacillus amyloliquefaciens subsp. *plantarum* MBI600 endospores. Our group has previously shown that MBI600 can interact with the plant host by triggering a signaling network that induces systemic plant defense responses (33), including resistance to viral pathogens of tomato (34).

In this study, the ability of *B. amyloliquefaciens* MBI600 to produce and secrete the catecholate siderophore bacillibactin is demonstrated. In parallel, bactericidal efficacy evaluation experiments showed that iron starvation induced production of bacillibactin, which significantly enhanced, *in vitro* and *in planta*, the bactericidal activity of MBI600 against the phytopathogenic species Pseudomonas syringae pv. *tomato*. It also broadened or enhanced the antibiotic activity against phytopathogenic fungi. Finally, it is shown that under iron starvation conditions, the expression of genes involved in bacillibactin biosynthesis is coordinated with that of genes involved in the synthesis of secondary metabolites as well as with antibiotic activity, cell mobility, and chemotaxis.

## RESULTS

### Characterization of siderophores that are secreted by *B. amyloliquefaciens* MBI600 under iron starvation.

**FIG 1:**
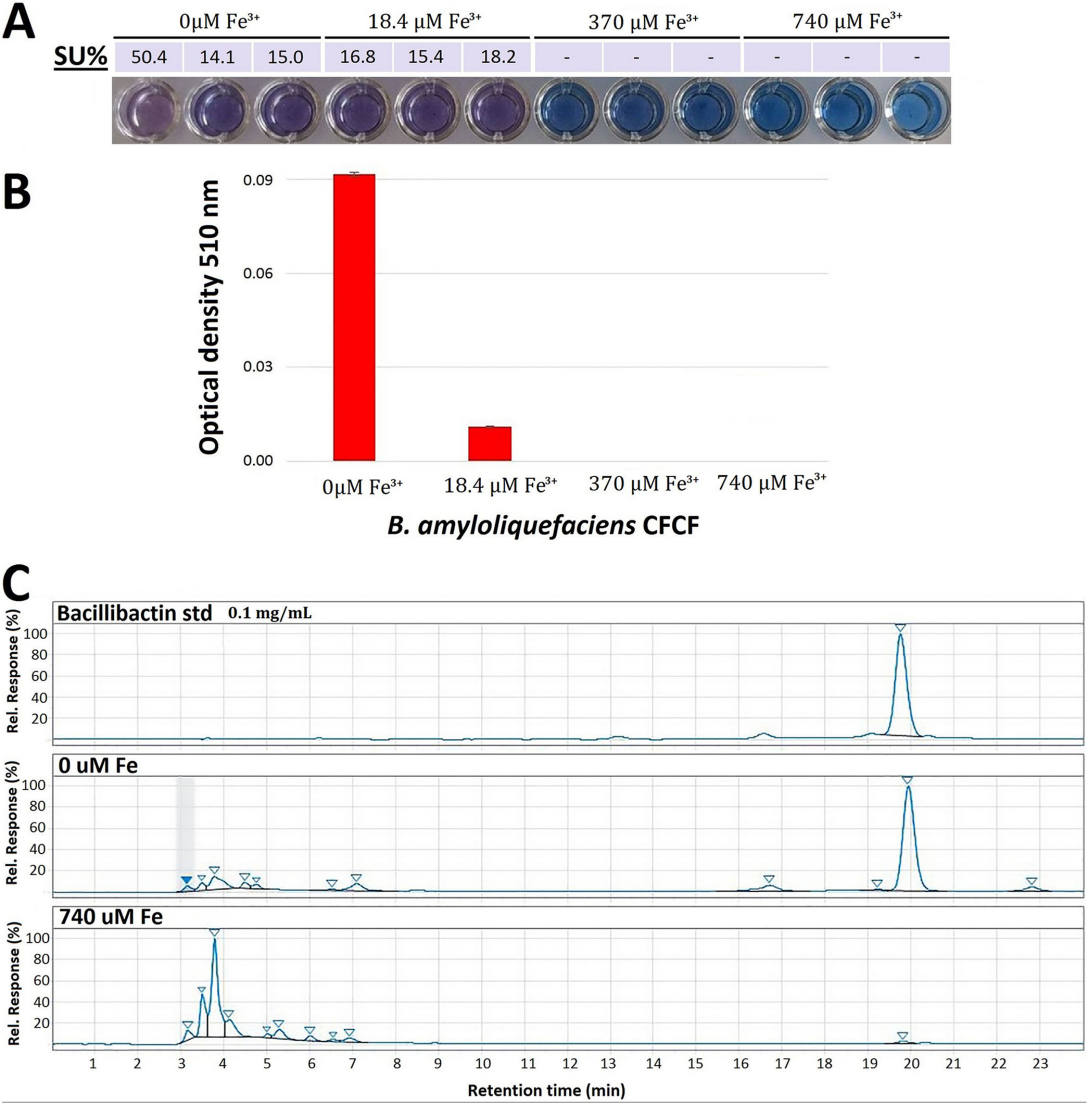
Production and identification of bacillibactin by *B. amyloliquefaciens* MBI600. (A) Chrome azurol S (CAS) liquid assay of MBI600 culture supernatants in increasing Fe^3+^ concentrations and respective siderophore unit (SU%) calculations. (B) Determination of the catecholate siderophore type by Arnow’s assay. (C) RP-HPLC chromatograms of bacillibactin commercial standard and siderophore extracts from MBI600 culture supernatants, in the Discovery BIO wide-pore C_18_-10 preparative column.

We screened for siderophore production in the supernatants of MBI600 cultures grown in chemically defined low-iron medium (CDLIM) (35) at various Fe^3+^ stress levels (0 and 18.4 μM added Fe^3+^ [iron starvation], 370 μM added Fe^3+^ [low iron stress], and 740 μM added Fe^3+^ [no iron stress]) using the chrome azurol S (CAS) assay (32). Under such iron stress conditions, MBI600 was capable of successfully growing (see Fig. S7 in the supplemental material). Siderophore production in the culture supernatants was clearly detected only under the iron starvation conditions of 0 and 18.4 μM Fe^3+^ (Fig. 1A). Catecholate groups, and the possible presence of bacillibactin, were identified using Arnow’s assay (increased absorbance at λ_max_ of 510 nm in the supernatants with 0 μM Fe^3+^), as shown in Fig. 1B.

10.1128/mSphere.00376-21.7 FIG S7 *B. amyloliquefaciens* MBI600’s growth curve under iron starvation conditions (0, 18.5, and 370 μM Fe^3+^). Optical densities at 600 nm were measured for 48 h at 37°C using a multidetection microplate reader and automatically recorded for each well every 29 min. Each ribbon depicts growth under a specific Fe^3+^ concentration; full line represents mean values and bars represent SEs from three independent biological experiments. Download FIG S7, EPS file, 0.5 MB. Copyright © 2021 Dimopoulou et al. 2021 Dimopoulou et al. https://creativecommons.org/licenses/by/4.0/ This content is distributed under the terms of the Creative Commons Attribution 4.0 International license.

Bacillibactin production in the culture supernatant under Fe^3+^ stress conditions (0 μM Fe^3+^) was verified through a two-stage reverse-phase high-performance liquid chromatography (RP-HPLC) analysis coupled with nuclear magnetic resonance (NMR) analysis. A clear peak at 315 nm and 19.72 min, corresponding to bacillibactin standard, was detected in the chromatograms of the first preparative column and only in the 0 μΜ Fe^3+^ supernatants (Fig. 1C). The peak was collected, concentrated, and applied in a second analytical column for further purification. A very clear peak was detected (see Fig. S1), which was collected for NMR analysis.

10.1128/mSphere.00376-21.1 FIG S1 HPLC chromatograms of the purified peak of bacillibactin from 0 μM Fe^3+^ supernatant and the commercial standard at 315 nm in the Poroshell 120 EC-C_18_ analytical column. (Inset) The corresponding NMR spectra. Download FIG S1, EPS file, 0.3 MB. Copyright © 2021 Dimopoulou et al. 2021 Dimopoulou et al. https://creativecommons.org/licenses/by/4.0/ This content is distributed under the terms of the Creative Commons Attribution 4.0 International license.

**FIG 2:**
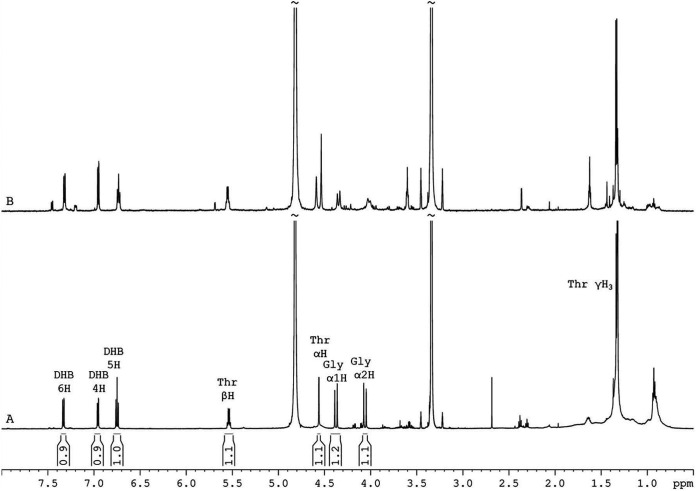
NMR analysis of the purified peak of 0 μM Fe^3+^ extract. Region (8.00 to 0.50 ppm) of ^1^H 1D NMR spectra of bacillibactin (BB) extracted from 0 μM Fe^3+^ supernatants of MBI600 (A) and commercially purchased bacillibactin (B) in deuterated methanol at 305 K. Peak integral values are noted between the *x* (ppm) axis and BB spectrum.

Following vacuum evaporation, the dried final peak was reconstituted in deuterated methanol for structure elucidation by ^1^H and ^13^C NMR spectroscopy. In the ^1^H one-dimensional (1D) NMR spectrum of the purified peak of 0 μM Fe^3+^ supernatant extract (Fig. 2A), all the characteristic resonances of bacillibactin structure were detected. The structure was further confirmed by a two-dimensional (2D) correlation spectroscopy (COSY) spectrum, where all the expected ^1^H-^1^H correlations were observed (Fig. S2A), and by a heteronuclear single quantum coherence-distortionless enhancement by polarization transfer (HSQC-DEPT) ^1^H-^13^C (Fig. S2B) spectrum, where protons are correlated with the corresponding carbon. All spectroscopic data, ^1^H and ^13^C chemical shifts, and ^1^H-^1^H and ^1^H-^13^C connectivities are in accordance with the literature (36, 37). For comparison reasons, the ^1^H 1D (Fig. 2B) and 2D NMR spectra were recorded also for the bacillibactin standard. ^1^H and ^13^C chemical shifts and coupling constants are provided in detail in Table 1.

**TABLE 1:** ^1^H and ^13^C chemical shifts of the bacillibactin extracted from 0 μM Fe^3+^ supernatants of MBI600^*a*^

Proton	^1^H shift^*b*^ (ppm)	Multiplicity*c* and coupling constant (Hz)^*c*^	^13^C shift^*d*^ (ppm)
DHB^*e*^ H-4	6.96	dd, 7.8/1.3	119.8
DHB H-5	6.75 t	t, 8.0	119.9
DHB H-6	7.33	dd, 8.1/1.4	119.6
Thr αH	4.56	d, 1.7	59.2
Thr βH	5.54	qd, 6.7/1.7	72.5
Thr γH	1.33	d, 6.6	17.3
Gly αH	4.38/4.06	d, 16.2	44.3

a The spectra were recorded in deuterated methanol (MeOD) (^1^H, 3.34 ppm; ^13^C, 49.15 ppm) at 305 K.

b ^1^H shift, 600.11 MHz.

c d, doublet; dd, doublet of doublets; t, triplet; qd, quartet of doublets.

d ^13^C shift, 150.9 MHz.

e DHB, 2,3-dihydroxybenzoic acid.

10.1128/mSphere.00376-21.2 FIG S2 ^1^H-^1^H COSY (8.00 to 0.50 ppm) (A) and ^1^H-^13^C HSQC-DEPT (8.00 to 0.50 ppm; 130 to 10 ppm) (B) NMR spectra of bacillibactin extracted from 0 μM Fe^3+^ supernatants of MBI600, in deuterated methanol, at 305 K. In HSQC-DEPT spectrum, methylene protons are colored red, while methine and methylene protons are black. Download FIG S2, EPS file, 0.1 MB. Copyright © 2021 Dimopoulou et al. 2021 Dimopoulou et al. https://creativecommons.org/licenses/by/4.0/ This content is distributed under the terms of the Creative Commons Attribution 4.0 International license.

### Iron starvation increases the susceptibility of P. syringae pv. *tomato* to MBI600.

**FIG 3:**
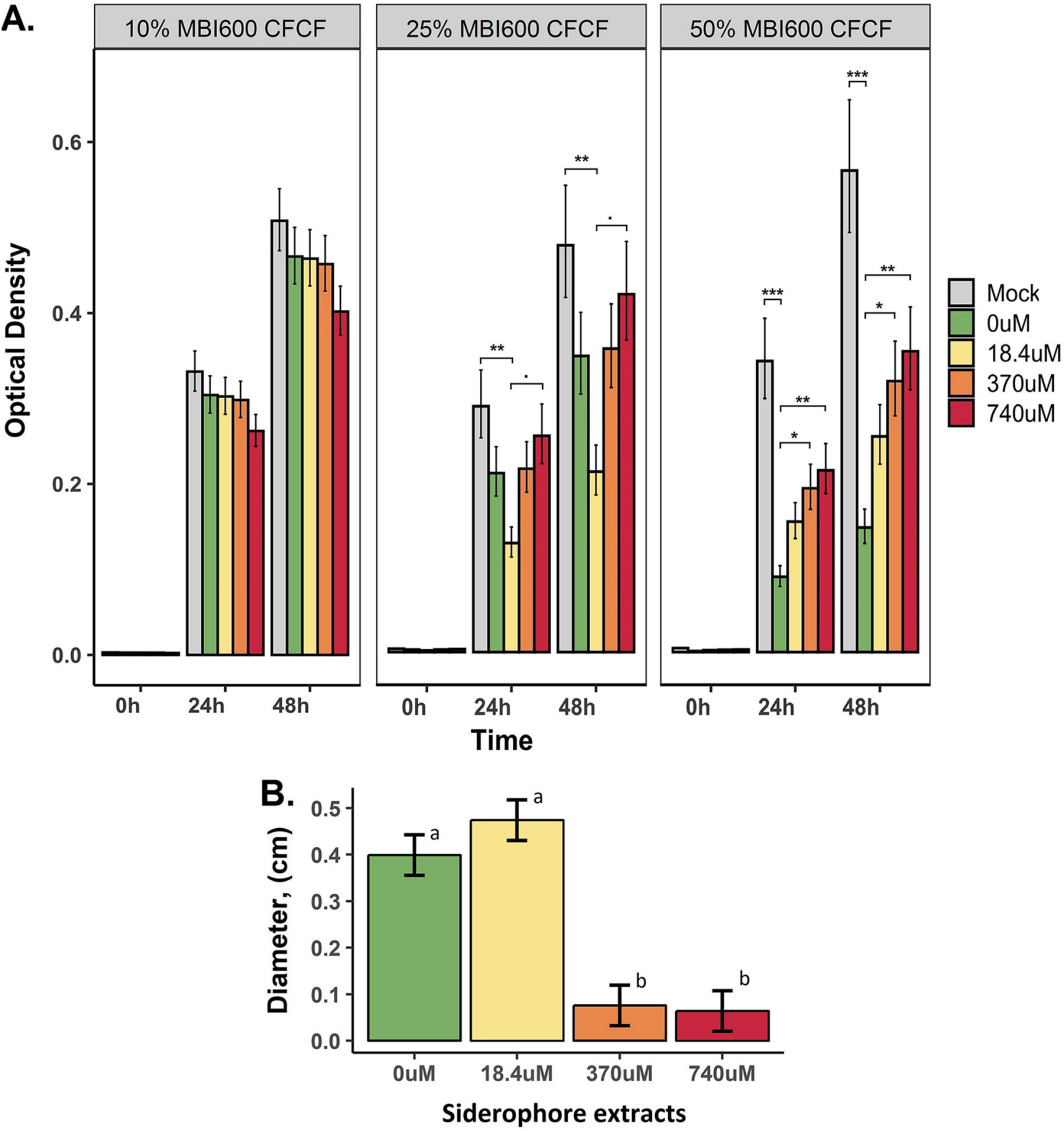
P. syringae pv. *tomato* becomes susceptible to MBI600 metabolites produced under iron starvation conditions. (A) *In vitro* bactericidal efficacy of CFCF. Susceptibility was tested against increasing MBI600 CFCF concentrations (0%, 2%, 10%, 25%, and 50% [vol/vol]). CFCF was extracted from MBI600 cells grown in a siderophore-producing medium (CDLIM) supplemented with increasing Fe^3+^ concentrations (0, 18.4, 370, and 740 μM) for 48 h. Observed effects were evaluated based on optical density at 600 nm (OD_600_) readings at 0, 24, and 48 hpi in microtiter plates. Estimated marginal means and their standard errors are plotted. ·, *P* < 0.1; *, *P* < 0.05; **, *P* < 0.01; ***, *P* < 0.001. (B) Inhibitory activity of bacillibactin. Estimated marginal means and standard errors of inhibition zone measurements in P. syringae pv. *tomato* agar cultures are plotted. Different lowercase letters represent statistically different estimates at a *P* value of ≤0.05 according to Tukey’s *post hoc* comparisons.

**(i) *In vitro* bactericidal efficacy of cell-free culture filtrate.**P. syringae pv. *tomato* was found to be marginally nonsusceptible to MBI600 in broth and plate susceptibility testing (see Fig. S3). Furthermore, it has been reported to assimilate xenosiderophores (38). It was, therefore, selected to assess the antibacterial activity of bacillibactin. P. syringae pv. *tomato* susceptibility was evaluated in dose-response treatments of MBI600 cell-free culture filtrate (CFCF) extracted from cultures grown under a range of iron starvation conditions (0, 18.4, 370, or 740 μM Fe^3+^) (Fig. 3A). P. syringae pv. *tomato* growth was not affected by the presence of 10% MBI600 CFCF in the case of all Fe^3+^ conditions and at both time points (24 and 48 h). It was only negatively affected by the 25% treatment with MBI600 CFCF extracted from iron-limiting conditions (0 μM and 18.4 μM Fe^3+^). However, under all iron conditions, except 18.4 μΜ Fe^3+^ (*P* = 0.0865 and 0.0102 for control and mock, respectively), results were not statistically significant compared to those for control (740 mM) and mock (LB) conditions, at both time points tested (Fig. 3A). The 50% CFCF treatment significantly decreased P. syringae pv. *tomato* growth in comparisons between MBI600 iron starvation conditions and control (0 μΜ Fe^3+^, *P* = 0.002) or mock (0 μΜ Fe^3+^, *P* < 0.0001; 18.4 μΜ Fe^3+^, *P* = 0.012) conditions. The effect of iron starvation was also confirmed in experiments using a different carbon source (succinic acid) (see Fig. S4) to exclude medium interference. In this case, there was a significant delay in P. syringae pv. *tomato* growth in the presence of 50% CFCF from MBI600 grown in iron-depleted broth compared to that grown in the iron-containing broth. Finally, iron starvation increased MBI600 bactericidal efficacy in agar plates containing a P. syringae pv. *tomato* lawn (see Fig. S5). The inhibition zone formed around MBI600 colonies was negatively correlated with the iron concentration of the agar medium.

10.1128/mSphere.00376-21.3 FIG S3 *In vitro* bactericidal efficacy of *B. amyloliquefaciens* MBI600. (Left) *P. syringae* pv. *tomato* broth susceptibility was tested against increasing MBI600 cell free culture filtrate (CFCF) concentrations (0, 0.5, 2, 10, 25, and 50% vol/vol). CFCF was extracted from MBI600 cells grown in LB medium. Observed effects were evaluated based on OD_600_ readings at 0, 24, and 48 hpi in microtiter plates. Estimated marginal means and their standard errors are plotted. (Right) No inhibition zone was formed around MBI600 culture grown on 9-mm Whatman discs that were placed on agar medium inoculated with *P. syringae* pv. *tomato*. Download FIG S3, EPS file, 1.4 MB. Copyright © 2021 Dimopoulou et al. 2021 Dimopoulou et al. https://creativecommons.org/licenses/by/4.0/ This content is distributed under the terms of the Creative Commons Attribution 4.0 International license.

10.1128/mSphere.00376-21.4 FIG S4 Effect on growth of Pseudomonas syringae pv. *tomato* (Pto) in LB medium supplemented with 10% (a), 25% (b), or 50% (c) *B. amyloliquefaciens* (Bam) CFCF (cell-free culture filtrate) supplemented with different iron concentrations (0, 18.4, 148, and 370 μM Fe^3+^). Optical densities at 600 nm were measured for 48 h at 28°C using a multidetection microplate reader. Estimated marginal means and their standard errors are plotted. Download FIG S4, EPS file, 2.4 MB. Copyright © 2021 Dimopoulou et al. 2021 Dimopoulou et al. https://creativecommons.org/licenses/by/4.0/ This content is distributed under the terms of the Creative Commons Attribution 4.0 International license.

10.1128/mSphere.00376-21.5 FIG S5 Inhibition zones formed in Pseudomonas syringae pv. *tomato* (Pto) agar cultures in the presence of *B. amyloliquefaciens* (Bam). Pto inoculum suspension was adjusted to 10^9^ CFU/ml and allowed to grow in agar medium containing increasing iron concentrations (0, 18.4, and 148 μM Fe^3+^). Values represent the averages of 24 inhibition zone measurements for each iron concentration. Different letters represent statistically different data points at a *P* value of ≤0.05 according to Tukey’s *post hoc* comparisons. Download FIG S5, EPS file, 0.7 MB. Copyright © 2021 Dimopoulou et al. 2021 Dimopoulou et al. https://creativecommons.org/licenses/by/4.0/ This content is distributed under the terms of the Creative Commons Attribution 4.0 International license.

### (ii) Bactericidal efficacy of siderophore extracts.

We tested the bactericidal efficacy of ethyl acetate siderophore extracts in agar cultures of P. syringae pv. *tomato*. Inhibition clear zones were observed and measured at 0.39 and 0.47 cm in the case of 0 and 18.4 μM Fe^3+^ conditions, respectively (Fig. 3B). When the iron supply was 370 or 740 μM Fe^3+^, that is, when bacillibactin was not detected, there was not a significant difference compared to the mock treatment (water, zero diameter). Both treatments leading to bacillibactin production (i.e., 0 and 18.4 μM Fe^3+^) led to statistically significant bactericidal efficacy compared to that with both 370 and 740 μM Fe^3+^ and controls.

### (iii) *In planta* bactericidal efficacy of CFCF.

**FIG 4:**
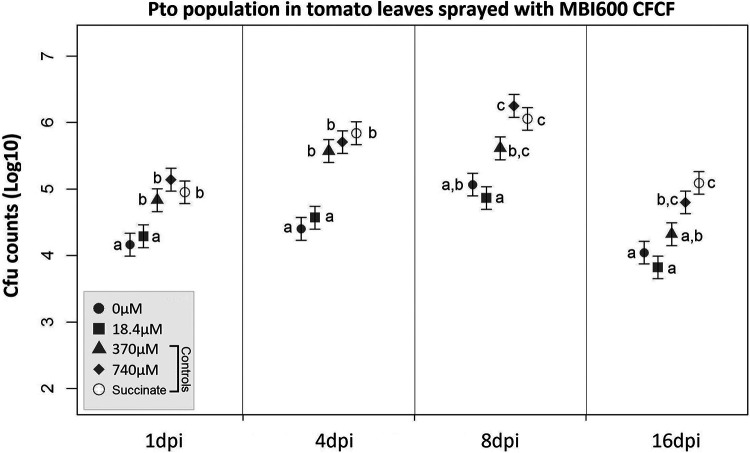
P. syringae pv. *tomato* population growth in tomato leaves sprayed with succinic acid medium (SM) (control) or CFCF of MBI600 grown in increasing Fe^3+^ concentrations (0, 18.4, 340, or 740 μM). Estimated means and corresponding standard errors of the logarithm of the bacterial population from 4 samples are shown. Different lowercase letters represent statistically different data points at a *P* value of ≤0.05 according to Tukey’s *post hoc* comparisons.

*In planta* bactericidal activity of CFCF was based on P. syringae pv. *tomato* total population in leaves of tomato plants sprayed with MBI600 CFCF over a period of 16 days postinoculation (dpi). At 1 dpi, the P. syringae pv. *tomato* population was significantly reduced after treatment with CFCF from iron-depleted media (0 and 18.4 μM Fe^3+^) compared to that in the controls (370 and 740 μM Fe^3+^ and the succinate control). Such differences were amplified at 4 dpi and persisted for several days before disease development (Fig. 4).

### Iron starvation broadens the antifungal activity of MBI600.

**FIG 5:**
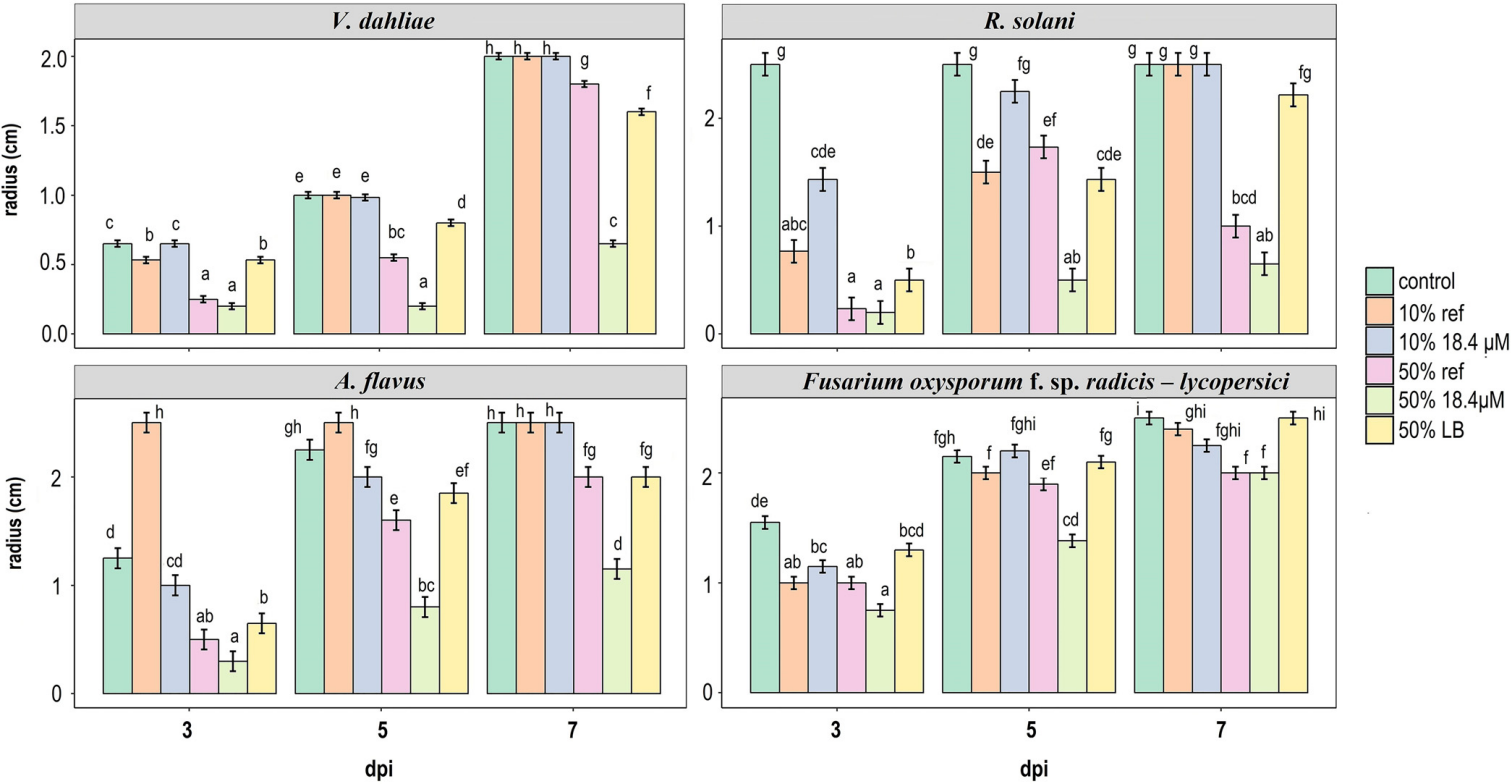
Iron starvation expands the range of antifungal activity of MBI600. Radial growth of 4 fungal species in minimal medium (MM) agar plates and in response to the following treatments: no treatment (control), 10% and 50% CFCF of MBI600 grown in 18.4 or 740 (ref) μM Fe^+3^ conditions, and 50% CFCF of MBI600 grown in LB. Values represent the estimated marginal means and corresponding standard errors of inhibition zone measurements. Different lowercase letters within each panel represent statistically different data points at a *P* value of ≤0.05 according to Tukey’s *post hoc* comparisons. Comparisons are among all treatments and among time points for each fungal species.

Based on the known antifungal range of MBI600 (Serifel label claims), we selected 7 strains of nonsusceptible fungal species (see Table S1) as well as Verticillium dahliae and oomycete Phytophthora cactorum strains, which are of intermediate susceptibility. Low susceptibility was confirmed using a treatment of MBI600 CFCF grown in LB (instead of the iron-limiting CDLIM) at the 50% (vol/vol) dose (nominated LB treatment). Susceptibility of all species was then tested against three doses/treatments (0, 10, and 50% [vol/vol]) of CFCF extracted from MBI600 cultures grown under 18.4 or 740 μΜ Fe^3+^ conditions as well as the LB treatment. Effects were attributed to bacillibactin only if comparisons between the 18.4 μM Fe^3+^ condition and each of the three controls (740 μΜ Fe^3+^ condition, no treatment, and LB treatment) were statistically significant. We found that iron starvation resulted in an inhibition of fungal growth, which was positively correlated with treatment dose and time (Fig. 5). Fungal growth was not affected by low CFCF doses (10%). On the other hand, all pathogenic species were found to be susceptible to the 50% treatment under the 18.4 μM Fe^3+^ condition. Growth inhibition was statistically significant in the cases of Fusarium oxysporum f. sp. *radicis*-*lycopersici* and Rhizoctonia solani at 5 dpi as well as in the cases of Aspergillus flavus and V. dahliae at 5 and 7 dpi (Fig. 5). In the cases of all other tested species, the 18.4 μM Fe^3^*^+^* condition showed statistically significant differences compared with control (0%) or LB treatments but not with 50% 740 μM Fe^3+^ (see Fig. S6).

10.1128/mSphere.00376-21.6 FIG S6 Iron starvation expands the range of antifungal activity of MBI600. Radial growth of 4 fungal species in minimal medium (MM) agar plates and in response to the following treatments: no treatment (control), 10% and 50% CFCF of MBI600 grown under either 740 μM Fe^+3^ (ref) or 18.4 μM Fe^+3^ conditions, and 50% CFCF of MBI600 grown in LB. Values represent the estimated marginal means and corresponding standard errors of inhibition zone measurements. ***, *P* < 0.001; **, *P* < 0.01; *, *P* < 0.05; ·, *P* < 0.1 according to Tukey’s *post hoc* comparisons. Download FIG S6, EPS file, 0.9 MB. Copyright © 2021 Dimopoulou et al. 2021 Dimopoulou et al. https://creativecommons.org/licenses/by/4.0/ This content is distributed under the terms of the Creative Commons Attribution 4.0 International license.

10.1128/mSphere.00376-21.8 TABLE S1 List of bacterial, fungal, and oomycete strains used in this study. Download Table S1, DOCX file, 0.1 MB. Copyright © 2021 Dimopoulou et al. 2021 Dimopoulou et al. https://creativecommons.org/licenses/by/4.0/ This content is distributed under the terms of the Creative Commons Attribution 4.0 International license.

### Determination of MBI600 gene expression in different iron concentrations.

**FIG 6:**
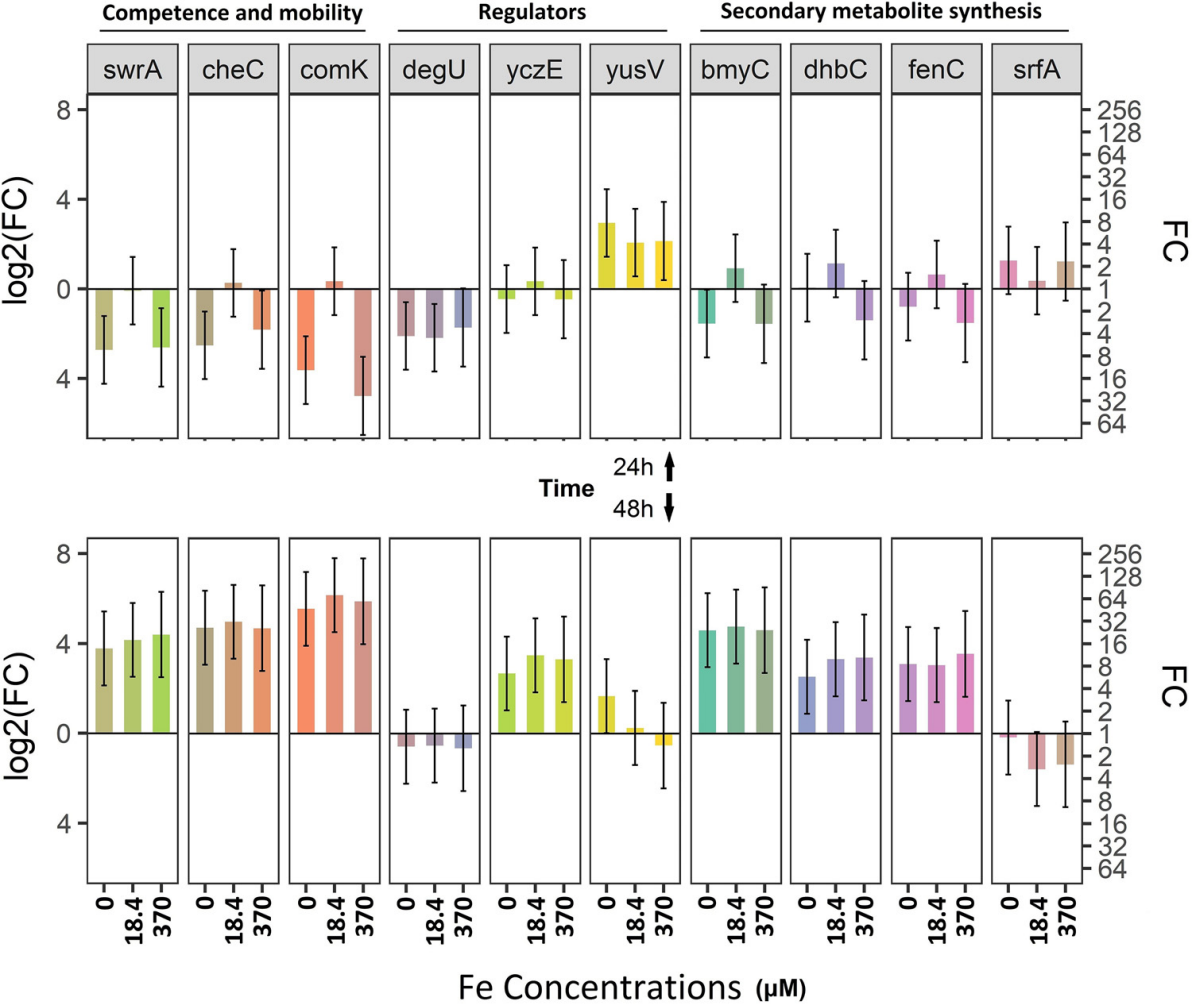
Iron starvation activates genes involved in MBI600 fitness. MBI600 cells were collected at 24 h (top) and 48 h (bottom) postinoculation of SM broth supplemented with increasing Fe^3+^ concentrations (0, 18.4, 340, or 740 μM). RT-qPCR analysis was performed using specific primers for the following genes: *dhbC*, siderophore synthase; *yusV*, siderophore transport system. Fold change (FC) represents the relative difference in expression between each treatment and control (740 μM Fe^3+^). *gyrA* was used as a reference gene. Bars indicate the 95% confidence interval (2 × standard error [SE]). Estimates whose confidence interval includes the baseline value 0 (1 in fold change scale) are not significantly different from control at an α of 0.05. All other differences are significant.

Using the aforementioned iron-depleted media, we showed that bacillibactin synthase (*dhbC*) is activated in iron-depleted cultures found in late static phase (48 h) but not under the 740 μM Fe^3+^ condition (Fig. 6), thus confirming siderophore production assays and the notion that 740 μM Fe^3+^ exceeds the threshold for iron deficiency (Fig. 1). Genes involved in *B. amyloliquefaciens* competence (*comK*), chemotaxis (*cheC*), and swarming (*swrA*) were significantly downregulated under all conditions compared to expression in the control at 24 h postinoculation (hpi). At 48 hpi, all 3 genes showed a significant upregulation under all iron conditions compared to expression in the control (baseline log2 fold change [FC]) (Fig. 6). A 2-fold upregulation of *srfAC* occurred at 24 hpi and in the cases of 0 and 370 μM Fe^3+^ conditions. Concerning the regulatory genes of these two groups of genes, those for competence and mobility, *yusV* was upregulated at 24 hpi under all iron conditions compared to expression in the control (740 μΜ Fe^3+^) and remained elevated at 48 hpi only in the case of the 0 μM Fe^3+^ condition. The upregulation of *yczE* occurred later (48 hpi) under all conditions compared to expression in the control, while the regulatory gene *degU* was not activated upon iron depletion.

## DISCUSSION

Bacillus amyloliquefaciens is known to produce a broad range of antimicrobial metabolites (22). Analysis of the *B. amyloliquefaciens* genome revealed 11 gene clusters, which occupy 9% of its genome, that are involved in the synthesis of antimicrobial metabolites (39). Three gene clusters are involved in nonribosomal synthesis of polyketides such as difficidin, macrolactin, and bacillaene, which are considered to have antibacterial activities (40, 41). Another five clusters are involved in nonribosomal synthesis of cyclic lipopeptides such as surfactin, bacillomycin, fengycin and bacillibactin (42). Surfactins are antibiotic compounds that show hemolytic, antimicrobial, and antiviral activities by altering membrane integrity (4, 43), whereas fengycin is specifically active against filamentous fungi and inhibits phospholipase A2 (44, 45). Bacillomycin D is a member of the iturin family, with a wide range of antibacterial and antifungal properties and hemolytic activities (26). Finally, bacillibactin is thought to contribute to the virulence of animal pathogens (46, 47), but its role in microbial competition remains to be elucidated.

Even if the role of siderophores in the biological control mechanism was first suggested 40 years ago (9), their antimicrobial activity has been reported only sporadically (48, 49). Siderophores have been studied for their roles in fitness and pathogenicity rather than biocontrol, with pyoverdine-producing pseudomonads being the model system (50). Lately, numerous studies correlate biological control and siderophore production by plant growth-promoting rhizobacteria (PGPRs). For example, Ghazy and El-Nahrawy suggested that two siderophore-producing strains, Bacillus subtilis MF497446 and Pseudomonas koreensis MG209738, reduced infection by Cephalosporium maydis in maize plant (51). Furthermore, Prema and Selvarani suggested microbial siderophores as potential biocontrol agents against plant pathogens (52). In this work, siderophore extract and bacillibactin were isolated from MBI600 cultures and tested for their bactericidal activity. This is the first report of bacillibactin isolation from commercial *B. amyloliquefaciens* species and characterization by NMR. We developed an HPLC protocol to isolate and purify bacillibactin from MBI600 culture supernatant, and its structure was confirmed by NMR analysis. All spectroscopic data were in accordance not only with the literature (33, 34) but also with our purchased bacillibactin standard (EMC Microcollections, Germany). Interestingly, NMR spectra revealed that isolated bacillibactin from this study showed higher purity than the purchased standard, highlighting the effectiveness of the developed isolation and purification protocol.

The combination of agar and broth tests suggested that P. syringae pv. *tomato* is susceptible to MBI600 cells and CFCF grown under iron-limited conditions. P. syringae pv. *tomato* inhibition was negatively correlated with the iron concentration of the MBI600 diet. Since MBI600 metabolites secreted in iron-rich media failed to inhibit Pseudomonas growth, it is concluded that iron starvation-elicited production of bacillibactin is responsible for this gain of function. This hypothesis was confirmed by the formation of inhibition zones around a bacillibactin extract in Pseudomonas-containing agar media. Production of siderophores was correlated not just with an extension of the biocontrol activity range of MBI600 against a nonsusceptible pseudomonad but also with biocontrol activity against nonsusceptible fungal pathogens. The strongest inhibition was observed in the cases of A. flavus, R. solani, and V. dahliae, while Botrytis cinerea, F. oxysporum f. sp. *radicis-lycopersici*, and *P. cactorum* growth was delayed in the presence of MBI600 siderophores. Results suggest that nutrition plays a crucial role in MBI600 antifungal activity, though *in planta* experiments are needed to confirm this notion. The fact that bacillibactin inhibited the growth of pathogens in iron-containing media suggests that its antibiotic function does not rely on iron scavenging to starve competing species. In this work, we identify, for the first time, the antibiotic potential of bacillibactin. Moreover, siderophores have been associated with iron scavenging (10). We suggest that their role in microbial competition should be reconsidered. Previous studies reported that several bacteria, including Pseudomonas, are able to utilize siderophores of other species (xenosiderophores) (38). It is, therefore, possible that bacillibactin is absorbed by Pseudomonas syringae pv. *tomato* and that its antibiotic function is intercellular.

Combined evidence supports the notion that to determine the range of biocontrol activity of BCAs *in vitro*, with the maximum correlation to *in planta* outcomes, it is necessary to test them in different minimal media in which both BCA and pathogens can grow. Such media would best mimic conditions of the nutrient-poor phyllosphere but also allow antagonists to fully exploit their antimicrobial arsenal. This hypothesis was confirmed *in planta*. Growth of MBI600 cultures under iron starvation conditions induced bacillibactin synthesis and secretion to a CFCF that was capable of reducing P. syringae pv. *tomato* population and delay the infection of tomato plants. Siderophores have already been hypothesized to contribute to the antifungal activity of B. subtilis *in planta* (10), but this is the first report that provides direct evidence of the antibacterial and antifungal activities of bacillibactin *in planta*.

The enhanced susceptibility of *Penicillium* sp. and Aspergillus niger to CFCF extracted from MBI600 cultures grown in both iron-depleted and iron-rich minimal media suggested that the nutrient composition activated the production of fungicidal metabolites in addition to bacillibactin. Expression analyses of (i) genes involved in secondary metabolite synthesis (*dhbC*, siderophore synthase; *bmyC*, bacillomycin [iturin] synthase; *yczE*, positive regulator of bacillomycin [iturin] synthesis; *fenC*, fengycin synthase; and *sfpAC*, surfactin) and (ii) *comΚ* (competition), *cheC* (chemotaxis), *swrA* (swarming), *deqU* (positive regulator of bacillomycin [iturin]), and *yusV* (probable siderophore transport system) confirmed the activation of the metabolic network responsible for *B. amyloliquefaciens* antibiosis, competence, and mobility.

It was previously shown that Bacillus subtilis has a strong antifungal activity due to the synergistic actions of surfactin, bacillomycin D, and fengycin (53, 54). Production of either of the latter two is articulately delayed (late stationary phase) compared with that of surfactin (transition between exponential and stationary growth) (55, 56). Accordingly, it was found that iron enhanced the expression of the surfactin biosynthetic gene *srfAC* at 24 hpi, while expression of bacillomycin synthase C (*bmyC*) and fengycin synthase C (*fenC*) was enhanced at 48 hpi. In support, *yczE*, a positive regulator of bacillomycin, was also activated at 48 hpi under low/depleted-iron conditions. These data suggest that MBI600 coordinately induces its offense to prevail against other organisms in iron-depleted habitats. Such a hypothesis is supported by the activation of *comK* during stationary phase, as previously reported (57, 58). In parallel, iron depletion was detected by chemotaxis and stimulated swarming motility toward more favorable conditions (59–61), as indicated by the overexpression of *cheC* and *swrA*, respectively. The fact that *degU*, which regulates motility and biofilm formation (62, 63), is not overexpressed under iron stress is expected, as its function is thought to depend on its protein phosphorylation (64). Expression of genes involved in biosynthetic pathways is an indication for the production of endpoint metabolites, but their actual levels can only be confirmed by quantification experiments. Production of bacillibactin was confirmed by activation of *dhbC*, an isochorismate synthase of the siderophore synthase operon (65), under iron depletion conditions at 48 hpi. In support, *yusV*, a gene involved in siderophore uptake (66), was overexpressed at 24 hpi in the cases of all treatments compared to expression with the reference treatment.

*B. amyloliquefaciens* MBI600 has been mostly used commercially against fungal diseases. This study confirmed that its bactericidal activity is broadened by iron starvation. The production of bacillibactin enhanced the biocontrol activity of MBI600. This is the first study showing that a member of *Bacillus* spp. can restrain P. syringae pv. *tomato* growth *in vitro* and *in planta*. Other studies showed that *B. amyloliquefaciens* restrained pathogen growth *in vitro* against other pseudomonads and *in planta* growth of Pseudomonas syringae pv *tomato*. In the case of the latter, biocontrol could rely in phylloplane competition or induction of host defenses (33, 34) rather than direct actions.

Evidence that elucidates the understanding of the elaborated arsenal of BCA antimicrobial metabolites could facilitate the improvement of biopesticides and their application methods. The former could be achieved by breeding BCA strains under specific conditions and utilizing novel active compounds produced. Enhancement of application methods can be achieved by combinational use of biopesticides with agents that might simultaneously enhance BCA aggression and pathogen susceptibility. Clinicians treat patients with transferrin-mediated iron sequestration therapy to maximize antibiotic impact and avoid the emergence of resistance (67). A similar approach could be adopted in agriculture with combinational use of chelating agents of fertilizers (e.g., EDTA) and biopesticides.

## MATERIALS AND METHODS

### Growth conditions of microorganisms.

*B. amyloliquefaciens* was routinely grown on tryptic soy agar (TSA; Oxoid) agar and tryptic soy broth (TSB; Oxoid) media at 37°C. Pseudomonas syringae pv. *tomato* was routinely grown on LB and nutrient agar (NA) media at 28°C for 48 h. Fungal and oomycete strains were grown on solid (12 g · liter^−1^ agar) minimal medium (MM) that consisted of salt solution supplemented with glucose (10 g · liter^−1^) and ammonium tartrate (1 g · liter^−1^) as carbon and nitrogen sources, respectively (68). The pH was adjusted to 6.8, and plates were incubated at 25°C. Bacterial species/pathovars and fungal and oomycete strains are listed in Table S1 in the supplemental material

### Siderophore production and purification.

All glassware (Borosil/Agarwal) used for growth medium and reagent preparation were cleaned, prior to use, with 6 N HCl to remove residual iron, rinsed with deionized water, and oven dried.

### (i) Growth conditions.

Chemically defined low-iron medium (CDLIM) consisted of (g · liter^−1^): KHSO_4_, 1.5; K_2_HPO_4_, 3; NaCl, 1; and NH_4_Cl, 5; pH 6.5. The medium was supplemented with 2 mg · liter^−1^ thiamine and 2% (wt/vol) glucose as an energy source (35). Finally, the following trace elements were added (mg · liter^−1^ in the final medium): CaCl_2_·2H_2_O, 100; MgSO_4_·7H_2_O, 80; ZnSO_4_·7H_2_O, 2; MnSO_4_, 0.0035; CuSO_4_, 0.005. CDLIM was further supplemented with various final Fe^3+^ concentrations (0, 18.4, 370, and 740 μM) using FeCl_3_. The MBI600 inoculum for siderophore production (2% [vol/vol] in the final culture) was prepared from 10 ml of an overnight culture in LB that was pelleted, washed once, and resuspended in 10 ml CDLIM. Flask cultures were incubated at 28°C and 200 rpm for 48 h. Cell mass was separated by centrifugation at 4,000 rpm for 15 min, and the supernatant was aspired for further treatment.

### (ii) Siderophore detection and type determination.

Siderophore production in the supernatant was detected by the chrome azurol S (CAS) method as described by Schwyn and Neilands (69) and quantitated in terms of percent siderophore units (70). Catecholate presence in the supernatants was performed through the Arnow’s test (71).

### (iii) Siderophore extraction.

A modified version of the siderophore extraction protocol developed by Patel et al. (35) was used. In brief, 200 ml of culture supernatant was acidified to pH 2.0 with 12 M HCl and extracted 2 times with equal volumes of ethyl acetate. Ethyl acetate fractions were pooled, concentrated to 2 ml using a rotary vacuum evaporator (Buchi), and stored at −20°C until further analysis.

### HPLC analysis of siderophore extracts.

Bacillibactin was quantified and purified from the various culture supernatants through reverse-phase HPLC on an Agilent 1220 Infinity LC system using a two-step purification protocol and an HPLC-grade bacillibactin standard (EMC Microcollections GmbH; Tubingen, Germany). The concentrated aqueous product from the ethyl acetate extraction was initially fractionated in a preparative column (Discovery BIO wide-pore C_18_-10, 25 cm by 10 mm, 10 μm; SUPELCO Analytical) in 250-μl batches and a 4.5-ml/min flowrate. The elution gradient was applied follows: 0 to 10 min, 100% solution A (sol.A) (acetonitrile [ACN]-H_2_O, 20:80, plus 0.1% trifluoroacetic acid [TFA]); 10- to 30-min linear gradient from 0% to 40% solution B (sol.B) (ACN-H_2_O, 80:20 plus 0.1% TFA). The principal sample peaks from the various runs that corresponded to bacillibactin standard (detection at 315 nm) were collected and concentrated to dryness in a rotary evaporator and resuspended in HPLC-grade methanol. The concentrate from the preparative column was further purified in 20-μl batches on an analytical column (Poroshell 120 EC-C_18_, 4 μm, 4.6 by 150 mm) at a flowrate of 0.8 ml/min. The elution gradient was applied as follows: 0 to 6.5 min with sol.A followed by a linear gradient (6.5 to 26.5 min) from 0% to 25% sol.B. Bacillibactin, eluted as a highly symmetrical peak, was collected, concentrated, and stored in HPLC-grade methanol at −20°C until NMR analysis.

### NMR analysis.

NMR spectra were recorded on a Bruker AVANCE III 600 spectrometer (Bruker, Karlsruhe, Germany) operating at 600.11 MHz for ^1^H and 150.9 MHz for ^13^C nuclei. Spectra were recorded at 305 K in deuterated methanol. The ^1^H 1D spectrum was recorded with analysis of 64,000 data points, for a spectral width of 10 ppm with 64 scans. The 2D COSY spectrum (pulse sequence cosygpqf, Bruker library) was recorded for a spectral width of 10 ppm with 2,000 data points in F2 dimension and 150 increments of 32 repetitions each. ^1^H-^13^C single bond correlations were detected in a HSQC-DEPT spectrum (hsqcedetgpsisp2.3, Bruker library) recorded with the standard parameters (^1^*J*_H-X_ = 145) for spectral widths of 12 ppm for ^1^H and 185 ppm for ^13^C nuclei, 2,000 data points in F2 dimension, and 150 increments of 32 repetitions each. The solvent signal was used for axis calibration. Data manipulations were performed with the software TopSpin v3.1 (Bruker, Germany).

### Cell-free culture filtrate preparation.

*B. amyloliquefaciens* MBI600 cells were allowed to grow in LB or siderophore-producing medium, CDLIM or succinic acid (72), for 24 or 48 h, respectively. CFCF was prepared as previously described (33). Siderophore production was higher in CDLIM than in the succinic acid medium that was initially used.

### Broth microdilution method.

The Pseudomonas syringae pv. *tomato* susceptibility test against *B. amyloliquefaciens* MBI600 was performed in 96-well microplates (Greiner CELLSTAR 96-well microplates, F-bottom) as previously described (73, 74). In brief, P. syringae pv. *tomato* was grown exponentially in overnight cultures and was streaked on LB plates to check its purity. Culture aliquots were then adjusted to reach a final concentration of 5 × 10^5^ CFU/ml, while MBI600 CFCF and LB medium concentrations were adjusted to reach final concentrations of 0, 0.5, 2, 10, 25, and 50% CFCF and 1× LB in a total volume of 100 μl in each well. Blank wells (treatments and medium only) containing each test concentration were also included in duplicates. Plates were incubated at 30°C for 48 h. Three independent biological experiments (inocula, CFCF extracts, and tested plates) were performed. Absorbance measurements were recorded at 0, 24, and 48 hpi at 30°C using triplicate readings from a multidetection microplate reader (Fluostar Galaxy; BMG Labtech) at 600 nm.

### Disk diffusion susceptibility tests.

To prepare dual cultures, 20 ml of overnight-grown naive stationary-phase cultures of each bacterial pathogen were adjusted to a concentration of 10^8^ CFU · ml^−1^ and added to 180 ml LB agar before the mixture was poured into petri dishes and incubated overnight at 30°C. A 40-μl MBI600 suspension, adjusted to 5 × 10^6^ cells, was then pipetted on 9-mm antibiotic assay discs (Whatman) that were placed on the agar cultures of each pathogen. As a control, sterile LB was used in place of the MBI600 suspension. Each plate was quadruplicated in 3 independent experiments and incubated at 30°C for 2 days before the diameter of the clear halo surrounding the disk was measured on three different axes. A similar protocol was developed to evaluate the effect of siderophore extracts, only this time, a 20-μl aliquot of concentrated siderophore extract was pipetted directly onto LB agar plates containing P. syringae pv. *tomato* pathogen suspension. As a control, a noninoculated liquid CDLIM for each Fe^3+^ concentration was used.

### Fungal susceptibility evaluation.

MBI600 CFCFs extracted from succinic acid broth or LB medium supplemented with increasing FeCl_3_ concentrations (18.4 or 740 μM Fe^3+^) were mixed with MM agar in ratios of 1:2 or 1:10 (vol/vol) and poured into petri dishes. MM agar was used as control. MM agar containing 10% or 50% MBI600 CFCF grown in minimal medium supplemented with 740 μM Fe^3+^ was used as the reference (ref). The technique of point inoculation was used for both treatment plates and controls (68). Each treatment was repeated three times. Plates were incubated at 25°C, and the radius of fungal growth was recorded daily on three different axes.

### *In planta* experiments.

Tomato plants were grown and inoculated with P. syringae pv. *tomato* as previously described (75). Plants were inoculated at the developmental stage of fully developed 4th true leaves. Pathogen inoculum was prepared from 5 ml overnight (18 h) cultures in 10 mM MgCl_2_ at a final concentration of 10^8^ CFU · ml^−1^. Two independent experiments were performed, each in 5 plots of 12 plants/plot. CFCF was harvested as aforementioned from MBI600 succinate broth cultures in which the Fe^3+^ concentration was adjusted to 0, 18.4, 370, or 740 μM Fe^3+^. Plain medium was used as a control. Pathogen inoculum and CFCF were simultaneously sprayed to run off using hand-pump sprayers and allowed to dry before sampling.

The pathogen population was estimated as previously described (75), based on cell counts in 4 pooled leaf disc samples, each containing 2 discs from 3 different plants per treatment. Samples were collected at 1, 4, 8, and 16 dpi from 4 upper leaves of plants at the 6 true leaf stage. Leaves were homogenized for 60 s with a tissue homogenizer (HCT Shaping System SA) in Bioreba extraction bags containing 10 ml of sterile distilled water. Aliquots of 0.1 ml from a 10-fold dilution series were plated on nutrient agar (NA) medium supplemented with 5 μg/ml rifampicin.

### Gene expression assays.

*B. amyloliquefaciens* MBI600 cultures were incubated in succinic acid medium with increasing FeCl_3_ concentrations (0, 18.4, 370, and 740 μM Fe^3+^) at 37°C and 200 rpm for 48 h. Total RNA was extracted from each sample using TRI reagent (Ambion) according to the manufacturer’s instructions. The concentration and purity of RNA were estimated using a P330 nanophotometer (IMPLEN). DNase-treated RNA (RNase-free DNase; New England BioLabs) was reverse transcribed into cDNA using Superscript II reverse transcriptase and oligo(dT) as primers (Invitrogen). Reverse transcription-quantitative PCR (RT-qPCR) was performed as previously described (33). Transcript levels of target genes were normalized relative to the level of *gyrA*. Primer sequences are listed in Table S2 in the supplemental material.

10.1128/mSphere.00376-21.9 TABLE S2 List of primers used in this study. Download Table S2, DOCX file, 0.1 MB. Copyright © 2021 Dimopoulou et al. 2021 Dimopoulou et al. https://creativecommons.org/licenses/by/4.0/ This content is distributed under the terms of the Creative Commons Attribution 4.0 International license.

### Data analysis.

**(i) Broth microdilution method.** Broth microdilution method analysis was performed using the method described in previous works (73, 74).

### (ii) Disk diffusion and mycelial growth susceptibility tests.

For the analysis of disk diffusion (bacterial) or mycelial growth (fungal) susceptibility, radial growth was modeled using linear mixed models (LMMs) in R language (76). In the case of bacterial diffusion susceptibility tests, before the estimation of marginal means, diameter estimates of the control assays (noninoculated liquid CDLIM) were subtracted from the experimental values. The corrected diameter was the dependent variable, and CFCF concentration was the independent fixed variable. For the fungal susceptibility tests, the radius of growth inhibition was the dependent variable, while CFCF concentration, time, and their interaction were the independent fixed variables. Repeated axial measurements nested within three replicated experimental plates were the random factors in both cases. Estimated marginal means and their standard errors were plotted, and Tukey’s *post hoc* comparisons were applied.

### (iii) Bacterial population assessment.

Pathogen growth *in planta* was assessed as previously described (75, 77). CFU counts were modeled using LMMs to log_10_-transformed data (number of bacterial CFU for each time point). The times after inoculation and treatment as well as their interaction were modeled as fixed factors, while plant individuals nested within a block comprised the random factor. Tukey’s tests were applied for *post hoc* comparisons of estimated marginal means.

### (iv) Gene expression data analysis.

RT-qPCR analysis was performed as previously described (33, 34). Threshold cycle (*C_T_*) values were modeled in the context of a LMM and comprised the independent variables, while time, gene, Fe concentrations, and their interactions were included as the dependent variables. Samples nested within experiments were the random factors of the model. Contrasts were formulated according to equation 5 reported by Steibel et al. (78), so that the reference gene (*gyrA*) and reference treatment (control, 740 μM Fe^3+^) represented the baselines of all relevant comparisons.
