# Supplementary material for: Direct Antibiotic Activity of Bacillibactin Broadens the Biocontrol Range of Bacillus amyloliquefaciens MBI600
Source: mSphere. 2021 Aug 11;6(4):e00376-21. doi: 10.1128/mSphere.00376-21 (PMC8386435; doi:10.1128/mSphere.00376-21)
Supplement: TABLE S1 [file msphere.00376-21-st001.docx]

| **Bacterial species** | **Strain** |
| --- | --- |
| *Pseudomonas syringae* pv. *tomato* (Pto) | DC 3000 |
| *Bacillus amyloliquefaciens* (Bam) | MBI600 |
| **Fungal species** | **strain** |
| *Aspergilus flavus* | CBS128202^a^ |
| *Aspergilus niger* | CBS513.88^a^ |
| *Botrytis cinerea* | Bc1 |
| *Fusarium oxysporum* f. sp. *radicis* - *lycopersici* | FRL1 |
| *Penicillium* | P1 |
| *Phytophthora cactorum* | ATHUM 5163^b^ |
| *Rhizoctonia solani* | AG2-1^c^ |
| *Sclerotinia sclerotiorum* | Ss1 |
| *Verticillium dahliae* | 70wt-r1^d^ |

*Galanopoulou et al., 2017

**deposited in the Culture Collection of Fungi of the Mycetotheca ATHUM

***personal collection of Dr E. Buttler, UC Davis, USA)

****kindly provided by Prof. Epaminondas Paplomatas, AUA, Athens, Greece
