# Supplementary material for: Direct Antibiotic Activity of Bacillibactin Broadens the Biocontrol Range of Bacillus amyloliquefaciens MBI600
Source: mSphere. 2021 Aug 11;6(4):e00376-21. doi: 10.1128/mSphere.00376-21 (PMC8386435; doi:10.1128/mSphere.00376-21)
Supplement: TABLE S2 [file msphere.00376-21-st002.docx]

| **Name** | **Forward primer (5'-3')** | **Reverse primer (5'-3')** |
| --- | --- | --- |
| *gyrA* | ACCGTAACGGAATGAGAATCGT | GCAGGGCCGTTTGTTTGTAC |
| *dhbC* | TGACCGCTGAAAAGGATATCG | TCACATCCGCAGCGAATGTA |
| *bmyC* | ACGGCTGCTGCAGATGCT | ACGGTCATAGACTTGTTCATTAAAACA |
| *yczE* | TCTTTCTGTCGGCCAATGGT | TCGGGAGTGTCCTCGTGAAC |
| *fenC* | TACACAGCTCCCCGCAATG | TGAGTCCTCAATCCCGACTTG |
| *srfAC* | GCATGAATGTGATTATGGACCG | TGTCCGTTACATGTGTCAGATCG |
| *comK* | GCCGATTCACATCATCGACA | ATGTGATTTGAAGGGTCCACCA |
| *cheC* | GAATGGAAGGCGACATGACC | AATATCAAATCCGGATTCCCG |
| *deqU* | GGTAGCAGAAGGTGACGATGG | CCTCTACACCATTTACATTCGGC |
| *yusV* | GAGAGCTCGCTATTTTGCCG | GCCACTGCGTCTTCGTCCT |
| *swrA* | ACGGATATTAAACGGTCCATGC | TCTCTTCCGTCATCCACAACG |
